# A Novel Open Tubular Capillary Electrochromatographic Method for Differentiating the DNA Interaction Affinity of Environmental Contaminants

**DOI:** 10.1371/journal.pone.0153081

**Published:** 2016-04-07

**Authors:** Lucia D’Ulivo, Yong-Lai Feng

**Affiliations:** Exposure and Biomonitoring Division, Environmental Health Science and Research Bureau, Environmental and Radiation Health Sciences Directorate, Health Canada, Ottawa, Canada; NSYSU, TAIWAN

## Abstract

The interaction of chemicals with DNA may lead to genotoxicity, mutation or carcinogenicity. A simple open tubular capillary electrochromatographic method is proposed to rapidly assess the interaction affinity of three environmental contaminants (1,4-phenylenediamine, pyridine and 2,4-diaminotoluene) to DNA by measuring their retention in the capillaries coated with DNA probes. DNA oligonucleotide probes were immobilized on the inner wall of a fused silica capillary that was first derivatized with 3-(aminopropyl)-triethoxysilane (APTES). The difference in retention times and factors was considered as the difference in interaction affinity of the contaminants to the DNA probes. The interaction of the contaminants with both double-stranded (dsDNA) and single-stranded DNA (ssDNA) coatings was compared. Retention factors of 1,4-phenylenediamine, pyridine and 2,4-diaminotoluene in the capillary coated with ssDNA probe were 0.29, 0.42, and 0.44, respectively. A similar trend was observed in the capillary coated with dsDNA, indicating that 2,4-diaminotoluene has the highest affinity among the three contaminants. The relative standard deviation (RSD) for the retention factors was in the range of 0.05–0.69% (*n = 3*). The results demonstrated that the developed technique could be applied for preliminary screening purpose to provide DNA interaction affinity information of various environmental contaminants.

## Introduction

The interaction between certain chemicals and DNA may lead to carcinogenicity [1,2]. Depending on their mode of interaction, these DNA binding chemicals can be categorized into classes of covalent and non-covalent binding. The latter includes intercalative binding and DNA major- and minor-groove binding. Therefore, measuring the interaction of environmental contaminants with DNA could provide important preliminary information about the potential carcinogenicity of the compounds.

Various methods have been utilized for analyzing the interaction of small molecular compounds with DNA. For example, a HPLC method in combination with an *in vitro* incubation process was used to study the DNA interaction with natural products [3]. However, the resolution for the separation of the DNA probe and interaction products was very poor. Recently, a rapid chromatographic method using HPLC and an *in vitro* system was developed to evaluate the interaction potencies and kinetic rates of reactive chemicals with ssDNA and dsDNA probes [4,5]. Although this method allows a quick screening of the interaction potencies of target chemicals, it needs an incubation of 48 h. Furthermore, the liquid-liquid extraction for quenching the unreacted chemicals is tedious and not eco-friendly.

Open tubular capillary electrochromatography (OT-CEC) is a new developing technique. Unlike packed CEC, in OT-CEC, the stationary phase is bonded to the inner wall of the capillary. Consequently, the preparation of OT-CEC columns is easier than packed CEC, and it avoids imprecision and containment problems of the stationary phase [6]. The column capacity of an OT-CEC capillary is also much less than a packed CEC capillary [7]. The OT-CEC technique mainly aims either to separate a mixture of analytes [8–11] or to study the interactions between the coating itself and target molecules [12–15]. The latter approach can be divided into either studies of specific interactions between a particular coating and an analyte of interest or studies of the coating *in situ* response to different environmental factors. This approach significantly simplifies the sample preparation without the need of any *in vitro* incubation. The flexibility of OT-CEC has allowed, in the last few years, the development of new columns with various types of coating materials [16,17]. In particular, a large number of studies were reported on where capillaries coated with DNA or RNA aptamers were used for recognizing and separating proteins, oligopeptides or small molecules [18–20]. However, so far, no attempt has been made to exploit capillary columns coated with oligonucleotides for screening of emerging pollutants. Furthermore, possible differences in the interaction affinity of the environmental contaminants with single-stranded and double-stranded oligonucleotides need to be assessed. The last but not the least, the production of aptamers is relatively expensive and time-consuming.

In this study, we developed a simple OT-CEC method to evaluate the interaction affinity of three target environmental contaminants, 1,4-phenylenediamine, pyridine, and 2,4-diaminotoluene, with DNA coated on capillaries. Phenylenediamines and diaminotoluenes are commonly used in the production of hair dye, textile fibers, agricultural chemicals and polyurethanes [21]. Pyridine is widely used as a solvent and intermediate in producing dyes, paints, rubbers and drugs [22]. Exposure from those contaminants has been studied to evaluate the risk of cancer formation [22,23].

In our study, 20-mer single-stranded and 20-bp double-stranded oligonucleotides were selected as coating material. Both retention times and retention factors were used for obtaining information on the degree of interaction affinity of the three contaminants with DNA. To the best of our knowledge, this is the first study to investigate the interaction affinities of those three contaminants to DNA with this technique. The developed method proved to be low-cost with a short screening time and a high sample throughput.

## Materials and Methods

### Chemicals

The ssDNA probes (RP1 purity, sequence 5’-3’) TGGACCTGGGCAATGTCTGG (20-mer, 5-HTT) and its complement CCAGACATTGCCCAGGTCCA (20-mer, RC-5-HTT were from Sigma-Genosys (Oakville, ON, Canada). Sodium phosphate monobasic (≥99%), sodium acetate (≥99%), sodium hydroxide (NaOH, ≥98), 3-(aminopropyl)-triethoxysilane (APTES, ≥98%), 1 M triethylammonium acetate (TEAA) buffer, glacial acetic acid (≥99.7%), acetone (anhydrous, 99.9% grade), dimethyl sulfoxide (DMSO, ≥99.5%), 1,4-phenylenediamine dihydrochloride (≥90%), pyridine (≥99.9%) and 2,4-diaminotoluene (98%) were purchased from Sigma-Aldrich (St-Louis, MO, USA). Hydrochloric acid (HCl, 36.5–38%) and methanol were from Omnisolv^®^ (EMD Millipore, USA). Deionized water (DIW) was made in-house with a Millipore Advantage system (Fisher Scientific, Ottawa, ON, Canada).

### Solution preparation

The 1 M NaOH solution was prepared by dissolving the appropriate amount of corresponding solid chemical in DIW. The two 10 mM phosphate buffer solutions (pH 7.4 and pH 6.5) were prepared by dissolving appropriate amounts of sodium phosphate in DIW and by adjusting their pH with 1 M NaOH solution. The 20 mM acetate buffer solution (pH 5) was prepared by dissolving an appropriate amount of sodium acetate in DIW and by adjusting its pH with 1 M acetic acid solution. The 10 mM TEAA buffer solution (pH 7) was prepared by diluting the 1 M stock solution in DIW. All the buffers were stored at 4°C.

The 500 μM stock solutions of 5-HTT and its complement were prepared by dissolving an appropriate amount of corresponding DNA probe in DIW, respectively, and stored at -20°C. The APTES working solution (2% v/v) was freshly prepared by diluting the APTES commercial solution in anhydrous acetone prior to use. The 10 mM stock solutions of three chemical, 1,4-phenylenediamine, pyridine and 2,4-diaminotoluene, were prepared by dissolving their corresponding solid compounds in DIW, respectively. The working mixture solution containing 0.5 mM of three chemicals was freshly prepared by diluting their 10 mM stock solutions with the 10 mM TEAA solution (pH 7) prior to sample analysis. The stock solution of 1% (v/v) DMSO was prepared by diluting an appropriate volume of DMSO in DIW.

### Preparation of dsDNA probes

The dsDNA probe (250 μM) was made in-house, according to the procedure reported by Gavina *et al*. [5]. Briefly, equimolar volumes (0.5 mL) of 500 μM 5-HTT solution and its complement were mixed in a 1 mL Eppendorf tube. The mixture was vortexed, then heated in a boiling water bath (1000 mL beaker) for 5 min, and then allowed to anneal by cooling naturally to room temperature in about 4 h. This synthesized dsDNA probe was then characterized with mass spectrometry. The prepared dsDNA probe solutions were store at -20°C until use.

### Coating of DNA probes on the capillary

An Agilent Technologies capillary electrophoresis (3D-CE, Agilent Technologies Inc., Waldbronn, Germany) equipped with a diode-array detector (DAD) and a thermostated cassette was used for the study. A fused bare silica capillary (Polymicro Technologies, Phoenix, AZ, USA, 50 μm inner diameter, 375 μm outer diameter) with a total length (L_tot_) of 38.5 cm and an effective length (L_eff_) of 30 cm was used for the study. The APTES coating procedure was modified from the method reported previously [24]. A new capillary was first preconditioned by flushing 10 min with 0.1 M NaOH, 10 min with DIW, 10 min with methanol and 20 min with anhydrous acetone at 25°C. Then, the cassette temperature was raised to 45°C. Subsequently, the APTES solution (2% v/v in anhydrous acetone) was infused into the capillary and maintained for 2 h to derivatize the inner surface of the capillary. After the derivatization, the capillary was flushed with anhydrous acetone for 20 min to remove the unreacted APTES solution. The cassette was then cooled down to 25°C and the capillary was immediately flushed with the 20 mM acetate buffer solution (pH 5) for 20 min to completely remove the acetone in the capillary. Then, the 0.2% DMSO water solution was injected under 50 mbar of pressure for 3 s. Subsequently, -20 kV of voltage was applied to run the injected DMSO out to check its migration time. The injection of the 0.2% DMSO water solution was repeated three times to evaluate the repeatability of the migration time as the reflection of the APTES coating stability. Only the capillary with a good APTES coating stability was then fully filled by infusing 250 μM of either ssDNA or dsDNA oligonucleotide probe solution under 50 mbar of pressure for 20 min. The solution was then kept in the capillary for another 20 min to let the DNA probe to be coated on the APTES surface. Then, the 20 mM acetate buffer (pH 5) was flushed through the capillary for 5 min to remove the DNA solutions. This DNA coating procedure was repeated twice more to let the APTES surface to be fully coated with the DNA probe. Finally, the capillary was flushed with the 10 mM phosphate buffer solution (pH 7.4) for 2 min. Similarly, 0.2% DMSO water solution was injected under 50 mbar of pressure for 3 s to measure the migration time of DMSO in the capillary coated with the DNA probe. The repeatability of the migration time for triplicate injections was also used to evaluate the stability of the DNA coating on the capillary surface.

### Interaction affinity measurement of contaminants to the DNA probe

1,4-phenylenediamine, pyridine and 2,4-diaminotoluene were used to demonstrate the applicability of the method on the measurement of their interaction affinities to the DNA probe. Prior to CE analysis, the capillary was flushed with the 10 mM TEAA solution (pH 7) for 2 min. TEAA was already successfully employed by Gavina *et al*. [5] to study the interactions between chemicals and DNA oligonucleotides. For comparison, TEAA was selected as separation buffer in our studies. Then, the freshly prepared working mixture solution containing 0.5 mM of the three chemicals was injected into the capillary by applying a positive voltage of 15 kV for 2 s. After the injection, a positive voltage of 20 kV was applied on the capillary to run the injected sample out. The capillary cassette temperature was set at 37°C. The UV wavelength was 200 nm with a bandwidth of 16 nm.

## Results and Discussion

### Derivatization of the fused silica capillary wall

**Fig 1:**
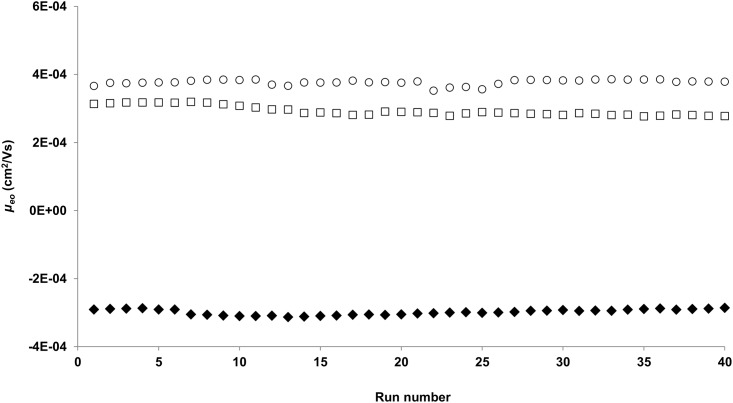
Stability test for APTES (filled diamonds), 20-mer ssDNA probe (void squares) and 20-bp dsDNA probe (void circles) coatings. The coating stability is given by the mobility (*μ*_*eo*_) of the EOF marker (DMSO) over 40 successive runs. Running conditions: injection 3 s 50 mbar, ±20 kV, 20 mM sodium acetate pH 5, 25°C. A negative polarity was used for APTES coating. The *μ*_*eo*_ of the bare fused silica column is 6.15E-04.

There are three types of surface coating techniques in OT-CEC, including dynamic, physically adsorbed, and covalent coatings. In this study, we used the covalent coating technique to obtain a positively charged surface. When a fused bare silica capillary is treated with a basic NaOH solution, its inner surface becomes negatively charged. In order to coat the negatively charged DNA oligonucleotide probes on the surface, the inner wall of the capillary has to be positively charged. The covalent derivatization of silica surfaces with APTES is commonly used to obtain a positively charged surface to enhance the adsorption of negatively charged molecules in capillary electrophoresis [24–26]. However, the incubation time in those reports was as long as 12 h. Waddel *et al*. subsequently improved the derivatization process to achieve the maximum stability of a silane-derivatized surface in a reduced incubation time (3 h) [26]. In this study, we investigated the influence of the derivatization time, from 0 to 12 h, on the stability of the APTES-derivatized surface. It was found that a derivatization time of 2 h was enough to provide a stable APTES coating for at least 40 runs (Fig 1). The RSD of the migration time of DMSO, the electroosmotic flow (EOF) marker, was 0.86% (*n = 10*) for a 2 h APTES-modified capillary. In contrast, a 12 h APTES-modified capillary provided a RSD value of 2.09% (*n = 10*), which is almost three times the RSD obtained for a 2 h APTES-modified capillary. This indicated that a coating incubation time longer than 2 h did not provide a more stable surface coating.

### DNA probe coating stability

**Fig 2:**
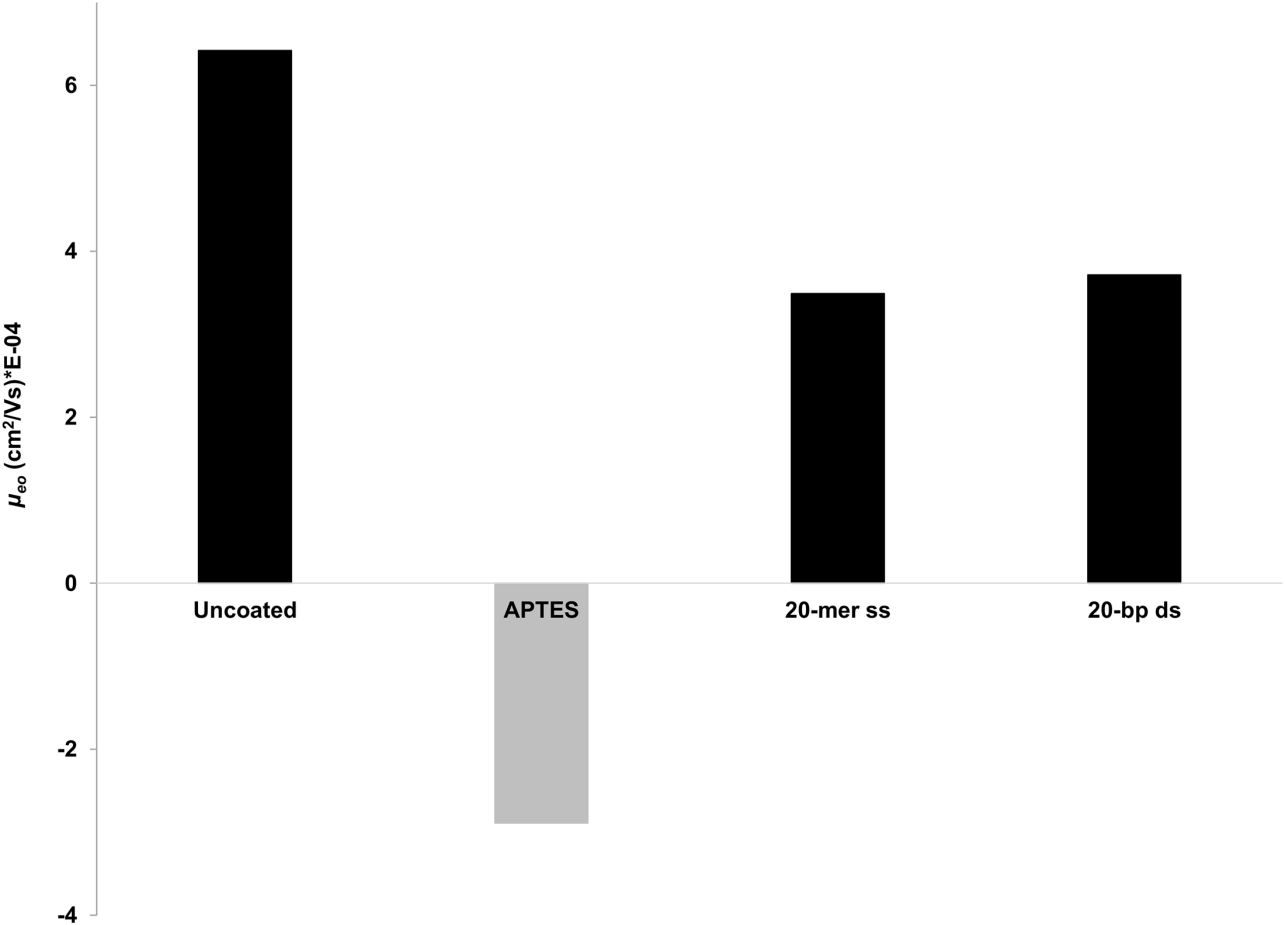
Change in the EOF mobility (*μ*_*eo*_) according to the coating type. DMSO was used as EOF marker. Running conditions: injection 3 s 50 mbar, ±20 kV, 10 mM sodium phosphate pH 7.4, temperature 37°C. A negative polarity was used for APTES coating.

When the inner surface of the capillary was successfully derivatized, the target DNA oligonucleotide probe was subsequently coated on the APTES surface. The stability of the DNA coating on APTES surface is crucial as it affects the repeatability of the sample analysis and the lifetime of the coated capillary. Therefore, we compared both stability and performances of the capillaries coated with either 20-mer ssDNA or 20-bp dsDNA oligonucleotides by testing the repeatability of the EOF mobility (*μ*_*eo*_) under different conditions. The 0.2% DMSO (EOF marker) water solution was injected under 50 mbar of pressure for 3 s, and 20 kV of positive voltage was applied to run DMSO out the capillary. The EOF mobility (*μ*_*eo*_) was then calculated with eq 1:

$$\mu_{eo}=\frac{L_{tot}L_{eff}}{Vt_{eo}}$$ (1)

where *L*_*tot*_ and *L*_*eff*_ are the total and the effective length of the capillary, respectively. *V* is the applied voltage, and *t*_*eo*_ is the migration time of the EOF marker, DMSO. We found that the direction of *μ*_*eo*_ was turned oppositely to that of the APTES capillary when the DNA oligonucleotides were coated on the APTES surface (Fig 2). This reverse turning of the EOF mobility indicated the successful coating of the DNA probe on the APTES surface. We also found that the value of EOF mobility for the capillary coated with ssDNA probe was close to the value of the capillary coated with dsDNA probe (Fig 2). This suggested that both ssDNA and dsDNA probes were able to provide a comparable coating performance for the capillary.

The repeatability of *μ*_*eo*_ over 40 successive runs was used as an indicator of good stability and life-time of the DNA probe coating. Fig 1 shows that both ssDNA and dsDNA probes were able to provide a stable coating surface for over 40 successive runs. The RSD of *μ*_*eo*_ over 40 successive runs were 4.7% and 2.2% for ssDNA and dsDNA coatings, respectively, indicating a good stability for both coatings. Furthermore, the RSD values of the DMSO migration time for 10 successive runs were used to quantitatively compare the stability of separation performance for the ssDNA and dsDNA probe coated capillaries. The RSD values were 2.42% and 1.37% for ssDNA and dsDNA probes, respectively, which suggested that both capillaries performed a stable migration in 10 successive run tests. It is important to underline that the stability test is indicative of how many consecutive runs can be performed with the same capillary. If the capillary is used discontinuously, for example, after three runs, the coated capillary is then kept with the buffer for a few days; this way may make bacterial growth in the capillary and alter the coated surface, leading into biased results. Therefore, we recommend using the coated capillaries in continuous mode to obtain the best performances.

The pH of the buffer was expected to influence the surface charges and the conformations of DNA probes. Therefore, it may influence the stability of the DNA coating on the APTES surface. However, our results indicated that the buffer pH had only little influence on the coating stability of the DNA probes. The coating with either ssDNA or dsDNA probe showed a very good stability in the pH range of 5–7.4 with RSD of 2.42–2.77% for *μ*_*eo*_ (*n = 10*). This indicated that the coatings with both ssDNA and dsDNA probes were applicable to broad working conditions. The influence of the cassette temperature on the coating stability was also investigated. The coatings were found to be stable in the temperature range of 25–37°C (RSD for *μ*_*eo*_ 2.52–5.89%, *n = 10*), suggesting that the coating was tolerable to relatively high working temperature.

### Interaction affinity of contaminants with DNA

**Fig 3:**
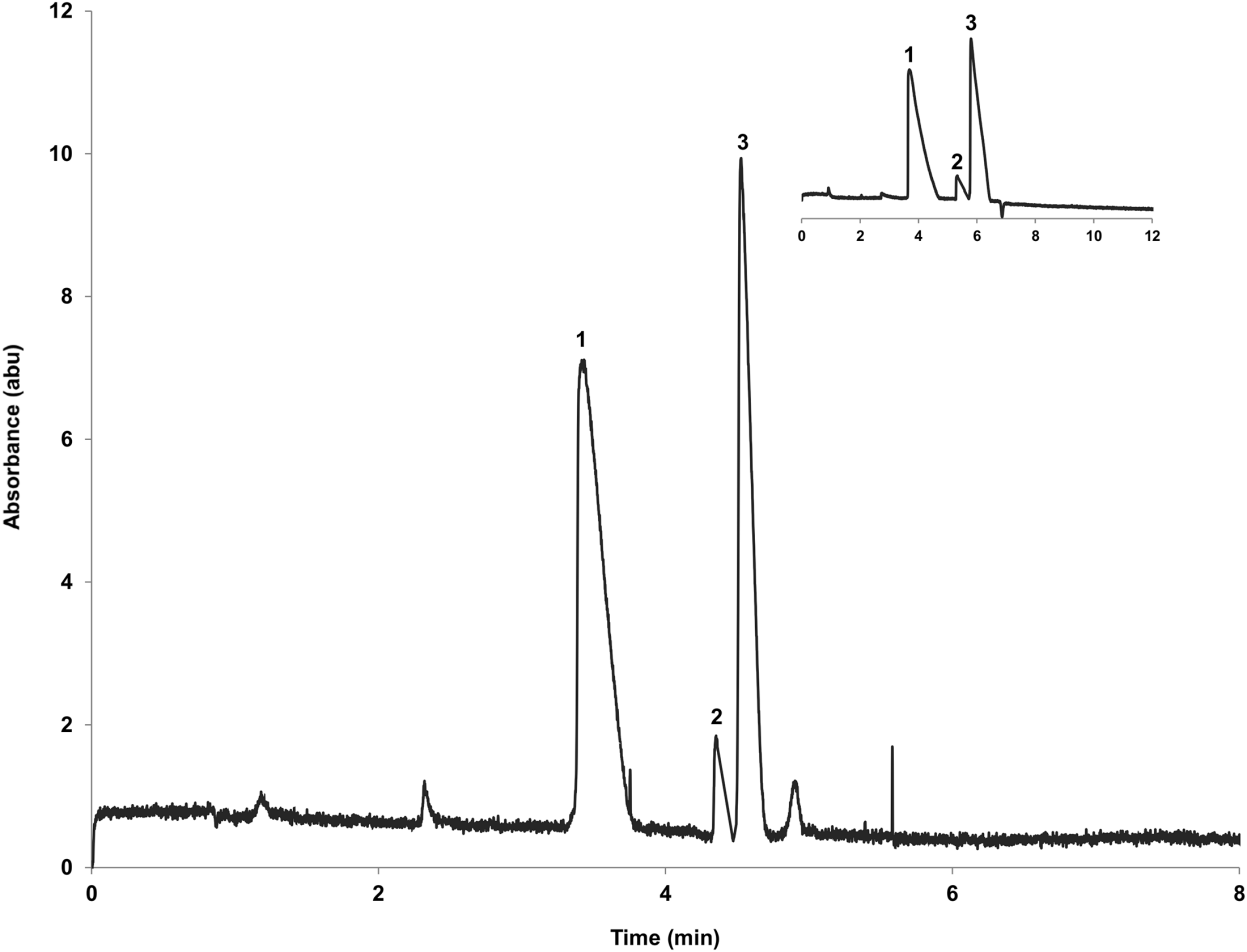
Separation of 1,4-phenylenediamine (1), pyridine (2) and 2,4-diaminotoluene (3) in 20-mer ssDNA probe and in 20-bp dsDNA probe (insert) coated capillaries. Sample concentration: 0.5 mM each in 10 mM TEAA pH 7. Electrokinetic injection for 2 s at +15 kV. Running conditions: +20 kV, 10 mM TEAA pH 7, 37°C. UV detection: 200 nm.

The interaction of a chemical with the immobilized DNA probe on the inner surface of the open tubular capillary will delay its movement in the running buffer. The stronger the interaction, the slower the movement will be. As a result, the chemical that has the strongest interaction with the DNA probe on the surface will need the longest migration time to move out the capillary. Therefore, the retention time on the chromatogram can reflect the interaction strength or the interaction affinity of the chemical. Alternatively, different retention times on chromatogram indicate interaction affinity difference of target chemicals to the DNA probes. The longer the retention time, the stronger the interaction affinity to the DNA probes will be expected. Fig 3 shows that the three model contaminants were baseline separated in the capillary coated with either ssDNA probe or dsDNA probe. 2,4-diaminotoluene had the longest retention time, pyridine was the next, and 1,4-phenylenediamine had the shortest retention time regardless of either ssDNA or dsDNA probe coating on the capillary (Fig 3). These results suggested that 2,4-diaminotoluene had the strongest interaction affinity, while 1,4-phenylenediamine the weakest.

In theory, the chromatographic retention factor of an analyte is a measure of the time the analyte resides in the stationary phase relative to the time it resides in the mobile phase. This factor can be also used to quantitatively evaluate the interaction of the analyte with the stationary phase. In CEC, the retention factor (*k*) of neutral/uncharged analytes can be calculated with eq 2 [27]:

$$k=\frac{t_{m}-t_{eo}}{t_{eo}}$$ (2)

where *t*_*m*_ and *t*_*eo*_ are the migration times of the analyte and of the EOF marker (the unretained compound), respectively.

However, for charged analytes in CEC, the above equation was previously modified as eq 3 [28,29]:

$$k^{\prime }=\left(1-\frac{v_{m}}{v_{m}+v_{eo}}\right)k-\frac{v_{m}}{v_{m}+v_{eo}}$$ (3)

Where *v*_*m*_ and *v*_*eo*_ are the velocities of the analyte and electroosmotic flow, respectively.

The *k’* values of the three contaminants in different capillaries coated with ssDNA and dsDNA probes are outlined in Table 1. The RSD values were less than 1% for triplicate runs, which indicated a good repeatability. We found that the *k’* values followed the order of 1,4-phenylenediamine < pyridine < 2,4-diaminotoluene for both ssDNA and dsDNA probe coated capillaries. These results suggested again that the interaction of 2,4-diaminotoluene with DNA probes was stronger than the other two contaminants. Pyridine and 1,4-phenylenediamine are classified in class 3 (not classifiable as to its carcinogenicity to humans), and 2,4-diaminotoluene was classified in class 2B (possibly carcinogenic to humans) by the International Agency for Research on Cancer (IARC) [22,30–31]. The binding affinities obtained in this study are in agreement with this IARC rank. Although pyridine is classified in the same group with 1,4-phenylenediamine, it exhibits higher affinity than 1,4-phenylenediamine with both ssDNA and dsDNA. This different behavior might be attributed to the distinctions on their structures and/or steric effects, indicating that molecular structure and amino group positions might influence the interaction affinity. We should underline that aromatic amines have intercalative properties that can play a crucial role in the interaction with the DNA coating. This property has been exploited by Wang. *et al*. for the development of a DNA biosensor for aromatic amines [32]. Wang noticed that the affinity of the aromatic amines was related to their structures and the position of the amino group, which was also previously reported by Tanius *et al*. [33]. The ratios of the retention factors for each contaminant in ssDNA probe coated capillary to dsDNA probe coated capillary were also calculated in Table 1. The ratios were all over 1, likely indicating that the interaction of three contaminants with ssDNA probe was stronger than with the dsDNA probe. This difference was also observed previously in the *in vitro* assay [5]. Interestingly, these ratios (*k’*_ss-DNA_/*k’*_ds-DNA_) followed the order of 2,4-diaminotoluene < pyridine < 1,4-phenylenediamine, which is opposite to the order of their *k’* values. This difference might suggest that the interaction affinity of chemicals to the dsDNA probe decreased faster than to the ssDNA probe. In other words, the resolution in the dsDNA probe coated capillary is slightly higher than in the ssDNA one. Therefore, the dsDNA probe coating was able to provide a better differentiation of the interaction affinity. This might be explained by the different conformations of dsDNA and ssDNA, which can provide different accessibility of chemicals to the DNAs.

**Table 1:** Retention factors of 1,4-phenylenediamine, pyridine and 2,4-diaminotoluene in capillary coated with 20-mer ssDNA probe (*k’*_*cec ssDNA*_) and 20-bp dsDNA probe (*k’*_*cec dsDNA*_).

	1,4-phenylenediamine	pyridine	2,4-diaminotoluene
***k’***_***cec ssDNA (RSD*, *n = 3)***_	0.29 (0.40%)	0.42 (0.61%)	0.44 (0.20%)
***k’***_***cec dsDNA (RSD*, *n = 3)***_	0.19 (0.69%)	0.34 (0.05%)	0.39 (0.61%)
$\frac{{k^{'}}_{cec\;ssDNA}}{{k^{'}}_{cec\;dsDNA}}$	1.52	1.24	1.14

In previous studies, DNA or RNA coated capillaries were used only for recognizing biomolecules and separating small molecules [18–20]. However, this study presents a different application on differentiating the interaction affinity of environmental contaminants to DNA in capillary electrophoresis. This could open a new direction on screening of DNA interaction affinity for emerging environmental contaminants for risk assessment. Both ssDNA and dsDNA probes provided the same performance in term of separation and retention of the contaminants, suggesting that this novel technique is a reliable method for differentiating the interaction affinity of environmental contaminants to DNA.

## Conclusions

In this study, an OT-CEC method was developed to differentiate DNA interaction affinity of environmental contaminants by coating either 20-mer ssDNA or 20-bp dsDNA probe on the capillary inner surface. The developed method was able to baseline separate three contaminants, namely 1,4-phenylenediamine, pyridine and 2,4-diaminotoluene. The interaction affinity to the DNA probes was represented by the retention time and retention factor of each contaminant on the capillary coated with DNA probes, which were in the order of 1,4-phenylenediamine < pyridine < 2,4-diaminotoluene. Compared to the ssDNA coated capillary, the dsDNA coated capillary can provide a better resolution, which suggests that the interaction between chemicals and ssDNA probes or dsDNA probes is likely associated to contaminant structures and DNA conformations. The repeatable results in the study demonstrated that the new coating technique was stable and reliable. It is anticipated that the proposed technique can find future application for screening studies of emerging environmental contaminants.
